# Simulation of Quantum Dynamics of Excitonic Systems at Finite Temperature: an efficient method based on Thermo Field Dynamics

**DOI:** 10.1038/s41598-017-08901-2

**Published:** 2017-08-22

**Authors:** Raffaele Borrelli, Maxim F. Gelin

**Affiliations:** 1 DISAFA, University of Torino, Grugliasco, I-10095 Italy; 2 Department of Theoretical Chemistry, Technische Universität München, Garching, D-85747 Germany

## Abstract

Quantum electron-vibrational dynamics in molecular systems at finite temperature is described using an approach based on Thermo Field Dynamics theory. This formulation treats temperature effects in the Hilbert space without introducing the Liouville space. The solution of Thermo Field Dynamics equations with a novel technique for the propagation of Tensor Trains (Matrix Product States) is implemented and discussed. The methodology is applied to the study of the exciton dynamics in the Fenna-Mathews-Olsen complex using a realistic structured spectral density to model the electron-phonon interaction. The results of the simulations highlight the effect of specific vibrational modes on the exciton dynamics and energy transfer process, as well as call for careful modeling of electron-phonon couplings.

## Introduction

Unraveling the role of quantum effects in the time evolution of various molecular systems and assemblies under realistic conditions at ambient temperatures is a key problem of modern physical and biological chemistry^1^. Long range energy and charge transfer in natural as well as in artificial systems are among the most important processes in which quantum coherent motion can be of relevance^2–5^. However, a proper understanding of these processes is often hampered by the impossibility to properly simulate the evolution of quantum systems with many degrees of freedom.

The quasi-adiabatic path integral (QUAPI)^6^ and the hierarchical equations of motion (HEOM)^7–9^ are among the most successful numerically exact methods for density matrix propagation. However, both methods become numerically demanding at low temperature^10^ and when the Hilbert space of the system is very large^8, 10–15^. A number of approximate methods based on density matrix formalism is also available, but their range of validity can be very limited and system dependent^16–25^. Numerically accurate evolution of large systems has also been described by the density matrix renormalization group (DMRG) methodology, and the associated time-evolution algorithms^26, 27^.

Wave function propagation methods employing a basis set representation, such as the multiconfiguration time-dependent Hartree (MCTDH) method and its multilayer extension (ML-MCTDH)^28, 29^, Gaussian based MCTDH and other basis set methods^30–33^, are powerful tools at very low temperature^34^, but become unhandy in high temperature cases, as their application requires a statistical sampling of the initial conditions and faces both theoretical and computational difficulties^35–37^. On the other hand, basis set methods are very versatile, and capable of handling a large variety of Hamiltonian operators^38, 39^.

The development of alternative approaches to the simulation of many body quantum dynamics at ambient conditions is therefore indispensable for better understanding and proper exploitation of quantum effects in nanosystems. In this work we discuss a novel theoretical methodology based on Thermo Field Dynamics theory^40, 41^ that combines an accurate Hamiltonian description of quantum dynamics at finite temperature with the flexibility of a basis set representation^42^. We then apply this methodology to the study of exciton dynamics in the Fenna-Matthews-Olsen (FMO) complex, which has nowadays become a “guinea pig” of exciton dynamics theory and quantum biology^1, 2, 43, 44^. The exciton dynamics at FMO has been simulated by all major numerically exact quantum methods, e.g. QUAPI^45^, HEOM^46, 47^, ML-MCTDH^48^. Several explicit parameterizations of the bath spectral density accounting for the impact of intra- and inter-molecular modes on the FMO dynamics have been developed^49, 50^. In the present work, we simulate exciton dynamics in FMO at ambient temperature modeling the electron-phonon interaction with a realistic spectral density obtained from experimental data^51^.

## Results

### Quantum Dynamics at Finite Temperature

Problems formulated in the quantum mechanics language require the calculation of the expectation value of some dynamical variable, *A*

$$\langle A(t)\rangle={\rm{T}}{\rm{r}}\{A(t)\rho (0)\}$$

where *ρ*(0) is the initial density matrix of the system, and *A*(*t*) = *e* ^*iHt*^ *Ae* ^−*iHt*^ is the Heisenberg representation of the operator *A*, *H* being the system Hamiltonian ($\hslash$ = 1). The trace operation implies a weighted sum over all the thermally accessible states. In most molecular systems the energies of the electronic degrees of freedom are usually much higher than the vibrational energies. The effect of a finite temperature is then to create a thermal population of excited vibrational states, while only one electronic state of the entire system, |*e*〉, is tangibly populated. Within the validity of this condition we can safely employ the approximation

1 $$\rho \mathrm{(0)}={Z}^{-1}{e}^{-\beta H}\approx |e\rangle \langle e|{\rho }_{{\rm{vib}}}.$$

Here *Z* is the proper partition function and *ρ*_vib_ is the equilibrium Boltzmann distribution of the vibrational degrees of freedom, which, in the present work, is described using harmonic approximation, and *β* = 1/*k*_*B*_ *T*, where *T* is the temperature of system and *k*_*B*_ the Boltzmann constant. Consequently, the trace operation involves only a summation over a thermal distribution of vibrational states, $|{n}_{1}{n}_{2}\ldots \rangle$

2 $$\begin{array}{rcl}\langle A(t)\rangle  & = & {Z}^{-1}\langle e|\sum _{{n}_{1}{n}_{2}\ldots }\,\langle {n}_{1}{n}_{2}\ldots |A(t){e}^{-\beta {\sum }_{k}{\omega }_{k}{a}_{k}{a}_{k}^{\dagger }}|{n}_{1}{n}_{2}\ldots \rangle |e\rangle \\  & = & {Z}^{-1}\langle e|\sum _{{n}_{1}{n}_{2}\ldots }\,\langle {n}_{1}{n}_{2}\ldots |{e}^{-\beta {\sum }_{k}{\omega }_{k}{a}_{k}{a}_{k}^{\dagger }/2}A(t){e}^{-\beta {\sum }_{k}{\omega }_{k}{a}_{k}{a}_{k}^{\dagger }/2}|{n}_{1}{n}_{2}\ldots \rangle |e\rangle .\end{array}$$

where ${a}_{k}^{\dagger },\,({a}_{k})$ are the creation (destruction) operators of the *k*-th bosonic degree of freedom with frequency *ω*_*k*_, and the cyclic invariance of the trace operation has been used for the symmetrization. Following the Thermo Field Dynamics approach^40^ the above trace can be evaluated by introducing a set of auxiliary boson operators ${\tilde{a}}_{k}^{\dagger },\,{\tilde{a}}_{k}$ and their corresponding occupation number states $|{\tilde{n}}_{1}{\tilde{n}}_{2}\ldots \rangle$, and rewriting the summation as^52^

3 $$\langle A(t)\rangle ={Z}^{-1}\langle e|\sum _{n}\,\langle {\tilde{n}}_{1}{\tilde{n}}_{2}\ldots |\langle {n}_{1}{n}_{2}\ldots |{e}^{-\beta {\sum }_{k}{\omega }_{k}{a}_{k}{a}_{k}^{\dagger }/2}A(t){e}^{-\beta {\sum }_{k}{\omega }_{k}{a}_{k}{a}_{k}^{\dagger }/2}|{n}_{1}{n}_{2}\ldots \rangle |{\tilde{n}}_{1}{\tilde{n}}_{2}\ldots \rangle |e\rangle .$$

The dummy tilde variables do not affect the expectation value since *A*(*t*) is independent of them, and the states $|\tilde{n}\rangle$ form a complete orthonormal set. We notice that in the above summation the numerical values of {*n*_*k*_} and $\{{\tilde{n}}_{k}\}$ are identical. Defining the so called thermal vacuum state

4 $$\begin{array}{rcl}|\mathrm{0(}\beta )\rangle  & = & {Z}^{-\mathrm{1/2}}\sum _{n}\,{e}^{-\beta {\sum }_{k}{\omega }_{k}{a}_{k}{a}_{k}^{\dagger }/2}|{n}_{1}{n}_{2}\ldots ,{\tilde{n}}_{1}{\tilde{n}}_{2}\ldots \rangle \\  & = & {Z}^{-\mathrm{1/2}}\,\exp \,(\sum _{k}\,{e}^{-\beta {\omega }_{k}/2}{a}_{k}^{\dagger }{\tilde{a}}_{k}^{\dagger })\,|0\rangle \\  & = & {e}^{-iG}|0\rangle \end{array}$$

where $|0\rangle =|0,\tilde{0}\rangle$ represent the vacuum state of the ensemble of physical and tilde bosons, and

5 $$G=-i\sum _{k}\,{\theta }_{k}({a}_{k}{\tilde{a}}_{k}-{a}_{k}^{\dagger }{\tilde{a}}_{k}^{\dagger }),\quad {\theta }_{k}={\rm{arctanh}}({e}^{-\beta {\omega }_{k}/2}),$$

the expectation value 3 becomes^53^

6 $$\langle A(t)\rangle =\langle e|\langle \mathrm{0(}\beta )|A(t)|\mathrm{0(}\beta )\rangle |e\rangle =\langle e|\langle 0|{e}^{iG}A(t){e}^{-iG}|0\rangle |e\rangle .$$

The above equation is an extension of the fundamental result of Thermo Field Dynamics^40, 54, 55^ and the transformation *e* ^−*iG*^ is often referred to as Bogoliubov thermal transformation^56^. Equation 6 can be equivalently written as (see Methods section, and ref. 42)

7 $$\langle A(t)\rangle =\langle \phi (t)|{A}_{\theta }|\phi (t)\rangle $$

where the wavefunction |*φ*(*t*)〉 satisfies the Schrödinger equation

8 $$i\frac{\partial }{\partial t}|\phi (t)\rangle ={\bar{H}}_{\theta }|\phi (t)\rangle ,\quad |\phi \mathrm{(0)}\rangle =|e\rangle |0\rangle $$

with the thermal operators

9 $${\bar{H}}_{\theta }={e}^{iG}\bar{H}{e}^{-iG}\quad {A}_{\theta }={e}^{iG}A{e}^{-iG}.$$

The modified Hamiltonian operator $\bar{H}$ is defined as

10 $$\bar{H}=H-{\tilde{H}}_{{\rm{vib}}}$$

where ${\tilde{H}}_{{\rm{vib}}}$ is any operator acting in the vibrational tilde space. Equations 7, 8, 9 and 10 are the main theoretical result of the work. In the methodology described above the evaluation of the thermal average 〈*A*(*t*)〉 can be reduced to the solution of the thermal Schrödinger equation 8 with the Hamiltonian ${\bar{H}}_{\theta }$ specified by Eq. 9, followed by the computation of the desired expectation value.

Since the coupling with the tilde space doubles the number of nuclear degrees of freedom, and since a thermal environment can be realistically mimicked only using hundreds or thousands degrees of freedom, the solution of the time-dependent Schrödinger equation 8 requires efficient numerical methods, suitable to treat a large number of dynamical variables^38^. Here we follow our recently proposed methodology^42^ and represent the full vibronic wavefunction using the so-called Tensor-Train (TT) format (Matrix Product States, MPS, in the physics literature)^57–59^. Equation 8 is then solved using a methodology based on the time-dependent variational principle (TDVP) recently developed by Lubich, Oseledets and Vandereycken^59^. The reader is referred to the original papers^58, 60^ for a detailed analysis of the TT decomposition, and to the Methods section (see also ref. 42).

### Exciton Dynamics in the FMO complex

In order to apply the above methodology to the study of exciton dynamics at finite temperature in the FMO complex we consider a widely employed model Hamiltonian in which a set {|*n*〉}, of coupled electronic states interacts linearly with a phonon bath

11 $$H=\sum _{n}\,{\varepsilon }_{n}|n\rangle \langle n|-\sum _{n\ne m}\,{J}_{nm}|n\rangle \langle m|+\sum _{k}\,{\omega }_{k}{a}_{k}^{\dagger }{a}_{k}-\sum _{kn}\,\frac{{g}_{kn}}{\sqrt{2}}|n\rangle \langle n|({a}_{k}^{\dagger }+{a}_{k}\mathrm{)}.$$

Here |*n*〉 describes an electronic state with the excitation localized on the *n*-th pigment, *ε*_*n*_ is the electronic energy of state |*n*〉, *J*_*nm*_ are electronic couplings, *ω*_*k*_ are the frequencies of the bath of harmonic oscillators, and the parameters *g*_*nk*_ determine the strength of the electron-phonon coupling. In the above notation the index *k* labels all the vibrations of the system.

The modified thermal Hamiltonian ${\bar{H}}_{\theta }$ which controls the finite temperature dynamics is readily obtained applying the Bogoliubov thermal transformation to the Hamiltonian operator 11^42^

12 $$\begin{array}{rcl}{\bar{H}}_{\theta } & = & {e}^{iG}\bar{H}{e}^{-iG}\\  & = & \sum _{n}\,{\varepsilon }_{n}|n\rangle \langle n|-\sum _{n\ne m}\,{J}_{nm}|n\rangle \langle m|+\sum _{k}\,{\omega }_{k}({a}_{k}^{\dagger }{a}_{k}-{\tilde{a}}_{k}^{\dagger }{\tilde{a}}_{k})\\  &  & -\sum _{kn}\,\frac{{g}_{kn}}{\sqrt{2}}\{({a}_{k}+{a}_{k}^{\dagger })\,\cosh \,({\theta }_{k})+({\tilde{a}}_{k}+{\tilde{a}}_{k}^{\dagger })\,\sinh \,({\theta }_{k})\}|n\rangle \langle n|.\end{array}$$

Here we have used ${\tilde{H}}_{{\rm{vib}}}={\sum }_{k}\,{\omega }_{k}{\tilde{a}}_{k}{\tilde{a}}_{k}^{\dagger }$ to exploit the invariance properties of the thermal Bogoliubov transformation^56^. At *T* → 0 the mixing parameters *θ*_*k*_ become zero, sinh (*θ*_*k*_) → 0, the coupling to the tilde space disappears, and the standard Schrödinger equation is recovered as expected. For high-frequency modes, ${\theta }_{k}\ll 1$, sinh (*θ*_*k*_) ≈ 0 and cosh (*θ*_*k*_) ≈ 1 even at room temperature. As a rule of thumb high-frequency modes need not be incorporated into the tilde Hamiltonian. This leads to additional reduction of the active space and computational savings.

The exciton part of the FMO Hamiltonian, the site energies *ε*_*n*_ and electronic couplings *J*_*nm*_, have been retrieved from ref. 61. Vibronic coherences are essentially determined by the distribution of the bath vibrational frequencies and their coupling constants *g*_*nk*_. Here we consider the general case in which the excited states of each BChl are independently coupled to a bath of *N* phonons (uncorrelated baths). Consequently, in the seven site FMO model, we have 7*N* vibrations, characterized by the coupling parameters *g*_*nk*_. Since a single pigment is excited in each electronic state, only *N* components of *g*_*nk*_ with $k=1+(n-\mathrm{1)}N,\ldots ,nN$ are nonzero for a given *n*. These parameters are assumed to be the same for all BChls and are conveniently specified by the so called bath spectral density^49, 50^

13 $$J(\omega )=\sum _{k}\,{g}_{k}^{2}\delta (\omega -{\omega }_{k}\mathrm{)}.$$

We also point out that in our methodology the values of the parameters *g*_*nk*_ can be determined using any method of choice, and no particular benefit is derived from the above specific assumption. Often the Drude-Lorentz spectral density is used, *J*(*ω*) = 2*γλω*/(*γ* ^2^ + *ω* ^2^), which however deviates substantially from the measured spectral density at 4 K^51^. Recent theoretical analyses suggest that the use of a structured spectral density can lead to quite relevant changes in the vibronic dynamics of the system^47, 48, 62, 63^.

Following the very recent work by Schulze *et al*.^48^, we model the electron-phonon interaction by discretizing the experimental spectral density of ref. 51 with *N* = 74 vibrations uniformly distributed in the range [2,300 cm^−1^]. This way the times corresponding to the frequency *ω*_*min*_ = 2 cm^−1^ and the line spacing Δ*ω* = (*ω*_*max*_ − *ω*_*min*_)/*N* are safely beyond the observed time evolution of the system. Accordingly, our model consists of 74 vibrations per molecular site, thus 518 overall vibrational degrees of freedom which are doubled to 1036 due to TFD methodology. The numerical approach to the solution of this problem is described in the Methods section.

**Figure 1:**
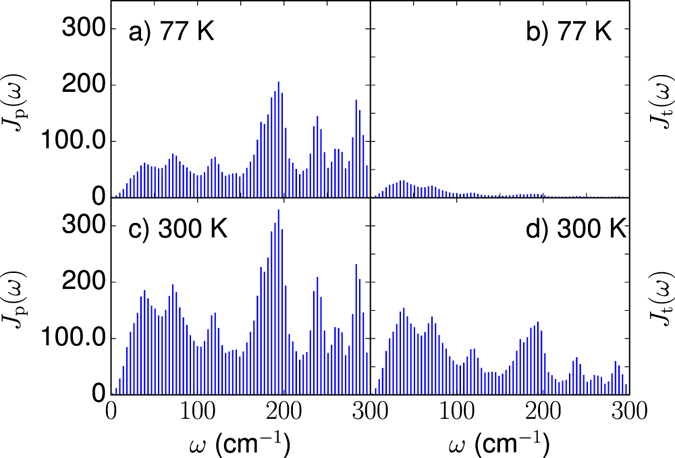
Effective site spectral densities *J*_*p*_(*ω*) and *J*_*t*_(*ω*) describing the coupling of the physical and tilde bosonic degrees of freedom with the electronic subsystem at different temperatures. (**a**,**b**) 77 K, (**c**,**d**) 300 K.

The parameters *g*_*kn*_ cosh (*θ*_*k*_) and *g*_*kn*_ sinh (*θ*_*k*_) entering the thermal Hamiltonian ${\bar{H}}_{\theta }$ govern the coupling of the electronic subsystem with physical and tilde bosonic degrees of freedom. Hence, it is tempting to introduce the spectral densities

14 $${J}_{p}(\omega )=\sum _{k}\,{({g}_{nk}\cosh ({\theta }_{k}))}^{2}\delta (\omega -{\omega }_{k}),\,{J}_{t}(\omega )=\sum _{k}\,{({g}_{nk}\sinh ({\theta }_{k}))}^{2}\delta (\omega +{\omega }_{k}),$$

which describe the electron-vibrational couplings in the physical (subscript *p*) and tilde (subscript *t*) subspace. As temperature goes to zero, *J*_*p*_(*ω*) → *J*(*ω*) and *J*_*t*_(*ω*) → 0. The two spectral densities are reported in Figure 1 at 77 K and 300 K. The comparison of lower and upper panels in Figure 1 reveals how effective electron-vibrational coupling increases with temperature, notably for lower-frequency modes.

**Figure 2:**
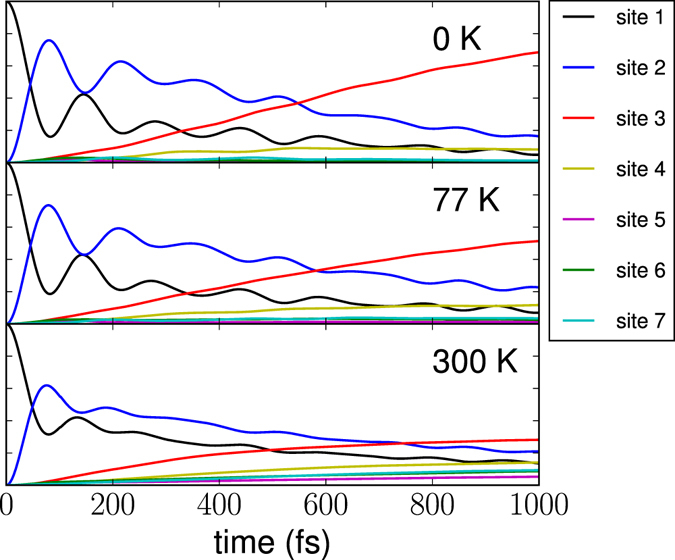
The time evolution of the electronic populations *p*_*n*_(*t*) of seven (*n* = 1–7) BChl_*a*_ molecules of the FMO complex at different temperatures indicated in the panels. The initial excitation is localized on site 1.

Figure 2 shows the total time-dependent populations *p*_*n*_(*t*) of seven (*n* = 1–7) BChl_*a*_ molecules of the FMO complex (standard numbering of the FMO cofactors is used). The populations are evaluated by Eqs 7 and 8 for *A* = *A*_*θ*_ = |*n*〉 〈*n*| so that *p*_*n*_(*t*) = 〈*A*(*t*)〉. The initial excitation is assumed to be initially localized on site 1. In all panels, *p*_1_(*t*) and *p*_2_(*t*) exhibit pronounced oscillations, as expected^45, 46^.

At *T* = 0 K (upper panel) the populations are in perfect agreement with the results obtained by Schulze and coworkers using ML-MCTDH^48^.

At *T* = 77 *K* (middle panel) *p*_3_(*t*) drops to about 0.6 at *t* = 1 ps. On the other hand, no pronounced difference in the behaviors of *p*_1_(*t*) and *p*_2_(*t*) at *T* = 0 and 77 K is observed. In the language of spectral densities defined in Eq. 14, it means that the contributions of the lower-frequencies vibrational modes (which are strongly temperature dependent) are quite significant in the dynamics of *p*_3_(*t*) already at 77 K, while are less pronounced in the dynamics of *p*_1_(*t*) and *p*_2_(*t*).

**Figure 3:**
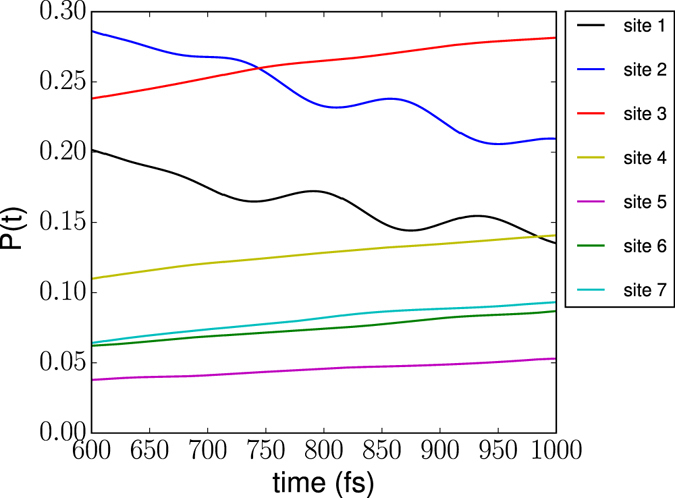
Enlarged section of the lower panel of Fig. 2.

If temperature increases up to 300 K (lower panel) *p*_3_(*t*) further decreases to 0.3 at *t* = 1 ps, and the oscillatory components of *p*_1_(*t*) and *p*_2_(*t*) are significantly reduced but still visible. As can be seen from Figure 3, which shows an enlargement of the lower panel of Figure 2 at longer times, small amplitude beatings of *p*_1_(*t*) and *p*_2_(*t*) are clearly observable even after 700 fs. Such long-lived beatings at ambient temperature have not been reported in models employing an approximate spectral density in the Drude-Lorentz form^46^, Ohmic form^45^ or Adolphs-Regner (single peak) form^45^. The beatings revealed in the present work at *T* = 300 K are due to the strongly structured spectral density and frequency-dependent coupling between the electronic subsystem and the vibrational degrees of freedom.

To elucidate how the spectral densities of Figure 1 affect the fraction of BChl_*a*_s that are significantly occupied during the time evolution of the system, we compute the inverse participation ratio Π(*t*), defined as^64, 65^

15 $${\rm{\Pi }}(t)=\frac{1}{{\sum }_{n}{p}_{n}^{2}(t)}.$$

**Figure 4:**
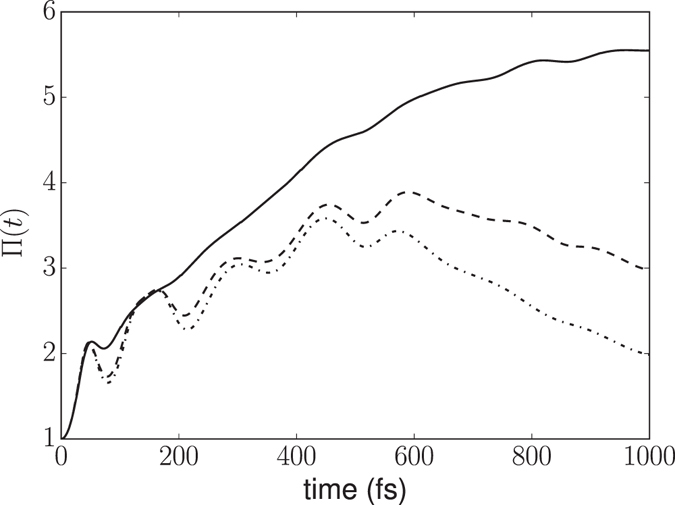
Inverse participation ratio Π(*t*) as a function of time; (−) 300 K, (− −) 77 K, (− ·) 0 K.

It is easy to show that Π(*t*) = 1 for a completely localized exciton wavefunction, while Π(*t*) = *N*_site_ (7 in the present case) for a perfectly uniform state. Therefore, Π(*t*) can be considered as an effective length, measuring the spatial extent of the exciton wave function over the aggregate. Figure 4 shows the computed Π(*t*) for the FMO complex at different temperatures. At *T* = 0 K Π(*t*) has a strong quantum behavior showing an oscillatory increase for the first 400 fs which is followed by an oscillatory decrease to a value of 2 at *t* = 1 ps. This is an indication of the exciton self-trapping. Therefore, a small number of sites are accessible to the systems during its evolution at *T* = 0 K, as is also evident from the population dynamics in Figure 2. For *T* = 77 K, the qualitative behavior of Π(*t*) remains the same but the number of accessible sites increases to 3 at *t* = 1 ps. At room temperature the number of accessible sites increases significantly and the effective length of the exciton is about 5.5 at *t* = 1 ps. The effect of a finite temperature is thus not only to provide a decoherence mechanism but also to increase the number of sites simultaneously accessible for the energy transfer process and to destroy the exciton self-trapping (cf. ref. 65).

Summarizing, we have developed a new theoretical and numerical approach for the determination of time dependent properties in large molecular aggregates. The methodology is based on Thermo Field Dynamics theory and requires doubling of all the thermalised degrees of freedom. The evaluation of an observable 〈*A*(*t*)〉 is reduced to the calculation of a simpler expectation value 〈*φ*(*t*)|*A*_*θ*_|*φ*(*t*)〉 where |*φ*(*t*)〉 satisfies the Schrödinger equation 8. The methodology has been implemented in the framework of the Tensor Train/Matrix Product State representation of the wave function, and using a novel technique for the numerical integration of Tensor Trains based on the time-dependent variational principle^59^. We have successfully applied this methodology to the study of quantum dynamics of energy transfer in the FMO complex. The present results draw attention to the importance of an accurate modeling of the bath spectral density. A highly structured spectral density is required to observe long-lasting oscillations in 〈*A*(*t*)〉 at room temperature which are absent when a simple Drude-Lorentz model^46^ or Ohmic model^45^ are employed.

The methodology developed in the present work offers new qualitative insights into the dynamics of excitonic systems at finite temperatures. The time evolution of these systems is governed by the thermal Hamiltonian ${\bar{H}}_{\theta }$ of Equation 12, which looks like a standard excitonic Hamiltonian *H* of Equation 11, but contains twice as many vibrational degrees of freedom: the physical ones and the auxiliary (tilde) ones. Hence, all nontrivial dynamic effects are governed by three sets of the parameters: the electronic couplings *J*_*nm*_ as well as the temperature-dependent electron-vibrational couplings *g*_*kn*_ cosh (*θ*_*k*_) and *g*_*kn*_ sinh (*θ*_*k*_). The parameters *g*_*kn*_ sinh (*θ*_*k*_) determine the coupling of the electronic degrees of freedom with the vibrational tilde degrees of freedom, and regulate the *effective number* of vibrational modes of the system. If *T* = 0 K, *g*_*kn*_ sinh (*θ*_*k*_) = 0 and the tilde variables are totally decoupled. At higher temperature both *g*_*kn*_ cosh (*θ*_*k*_) and *g*_*kn*_ sinh (*θ*_*k*_) tend to contribute on an equal footing. Since the system dynamics is purely Hamiltonian, the time evolution of any observable 〈*A*(*t*)〉 is the result of pure dephasing of the wave packet formed by a large number of vibronic eigenstates of the Hamiltonian ${\bar{H}}_{\theta }$. All oscillatory behaviors in 〈*A*(*t*)〉 are therefore vibronic by definition, since they are caused by the combined effect of electronic and temperature-dependent electron-vibrational couplings. Hence, a proper simulation of the time evolution of excitonic systems at finite temperature requires a careful modeling of electron-phonon interaction.

## Methods

### Derivation of TFD Schrödinger equation

Equation 6 can be transformed into a convenient Schrödinger representation by first rewriting it as

16 $$\begin{array}{rcl}\langle A(t)\rangle  & = & \langle e|\langle 0|{e}^{iG}{e}^{iHt}A{e}^{-iHt}{e}^{-iG}|0\rangle |e\rangle \\  & = & \langle e|\langle 0|{e}^{iG}{e}^{i(H-{\tilde{H}}_{{\rm{vib}}})t}A{e}^{-i(H-{\tilde{H}}_{{\rm{vib}}})t}{e}^{-iG}|0\rangle |e\rangle \end{array}$$

where ${\tilde{H}}_{{\rm{vib}}}$ is any operator acting in the tilde vibrational space. The choice of the *gauge* ${\tilde{H}}_{{\rm{vib}}}$ is dictated exclusively by computational convenience and does not affect the expectation value 〈*A*(*t*)〉. Hence

17 $$\langle A(t)\rangle =\langle e|\langle 0|{e}^{iG}{e}^{i(H-{\tilde{H}}_{{\rm{vib}}})t}A{e}^{-i(H-{\tilde{H}}_{{\rm{vib}}})t}{e}^{-iG}|0\rangle |e\rangle =\langle e|\langle 0|{e}^{i{\bar{H}}_{\theta }}{A}_{\theta }{e}^{-i{\bar{H}}_{\theta }t}|0\rangle |e\rangle .$$

where

18 $${\bar{H}}_{\theta }={e}^{iG}\bar{H}{e}^{-iG}\quad {A}_{\theta }={e}^{iG}A{e}^{-iG},$$

and the modified Hamiltonian operator $\bar{H}$ is defined as

19 $$\bar{H}=H-{\tilde{H}}_{{\rm{vib}}}.$$

Equation 17 is clearly equivalent to

20 $$\langle A(t)\rangle =\langle \phi (t)|{A}_{\theta }|\phi (t)\rangle $$

where the wavefunction |*φ*(*t*)〉 satisfies the equation

21 $$i\frac{\partial }{\partial t}|\phi (t)\rangle ={\bar{H}}_{\theta }|\phi (t)\rangle ,\quad |\phi \mathrm{(0)}\rangle =|e\rangle |0\rangle .$$

We point out that in order to obtain a numerical solution of the Schrödinger equation 21 the Hamiltonian ${\bar{H}}_{\theta }$ must have an analytical representation or a form which is suitable for numerical treatment. This can be accomplished by expanding $\bar{H}$ in power series of creation-annihilation operators (or position and momentum operators) and using the fundamental relations^40^

22 $${e}^{iG}{a}_{k}{e}^{-iG}={a}_{k}\,\cosh \,({\theta }_{k})+{\tilde{a}}_{k}^{\dagger }\,\sinh \,({\theta }_{k})$$

23 $${e}^{iG}{\mathop{a}\limits^{ \sim }}_{k}{e}^{-iG}={\mathop{a}\limits^{ \sim }}_{k}\,\cosh \,({\theta }_{k})+{a}_{k}^{\dagger }\,\sinh \,({\theta }_{k}).$$

The transformed Hamiltonian ${\bar{H}}_{\theta }$ depends on temperature through the parameters *θ*_*k*_.

### Quantum Dynamics with Tensor-Trains

Let us consider a generic expression of a state of a *d* dimensional quantum system in the form

24 $$|{\rm{\Psi }}\rangle =\sum _{{i}_{1},{i}_{2},\ldots ,{i}_{d}}\,C({i}_{1},\ldots ,{i}_{d})|{i}_{1}\rangle \otimes |{i}_{2}\rangle \cdots |{i}_{d}\rangle .$$

where |*i*_*k*_〉 labels the basis states of the *k*-th dynamical variable, and the elements $C({i}_{1},\ldots ,{i}_{d})$ are complex numbers labeled by *d* indices. If we truncate the summation of each index *i*_*k*_ the elements $C({i}_{1},\ldots ,{i}_{d})$ represent a tensor of rank *d*. The evaluation of the summation 24 requires the computation (and storage) of *n* ^*d*^ terms, where *n* is the average size of the one-dimensional basis set, which becomes prohibitive for large *d*. Using the TT format, the tensor *C* is approximated as

25 $$C({i}_{1},\ldots ,{i}_{d})\approx {G}_{1}({i}_{1}){G}_{2}({i}_{2})\cdots {G}_{d}({i}_{d})$$

where *G*_*k*_(*i*_*k*_) is a *r*_*k*−1_ × *r*_*k*_ complex matrix. In the explicit index notation

26 $$C({i}_{1},\ldots ,{i}_{d})=\sum _{{\alpha }_{0}{\alpha }_{1}\cdots {\alpha }_{d}}\,{G}_{1}({\alpha }_{0},{i}_{1},{\alpha }_{1}){G}_{2}({\alpha }_{1},{i}_{2},{\alpha }_{2})\cdots {G}_{d}({\alpha }_{d-1},{i}_{d},{\alpha }_{d}\mathrm{)}.$$

The matrices *G*_*k*_ are three-dimensional arrays, called *cores* of the TT decomposition. The ranks *r*_*k*_ are called compression ranks. Using the TT decomposition 25 it is possible, at least in principle, to overcome most of the difficulties caused by the dimensions of the problem. Indeed, the wave function is entirely defined by *d* arrays of dimensions *r*_*k*−1_ × *n*_*k*_ × *r*_*k*_ thus the required storage dimension is of the order *dnr* ^2^.

In a time-dependent theory the cores *G*_*k*_(*i*_*k*_) are time dependent complex matrices whose equations of motion can be found by applying the time-dependent variational principle (TDVP) to the parametrized form of the wave function

27 $$|{\rm{\Psi }}(G(t))\rangle =\sum _{{i}_{1}\cdots {i}_{d}}\,{G}_{1}({i}_{1},t){G}_{2}({i}_{2},t)\cdots {G}_{d}({i}_{d},t)|{i}_{1}\rangle \otimes |{i}_{2}\rangle \cdots |{i}_{d}\rangle .$$

The resulting equations of motion can be written in the form

28 $$\frac{d}{dt}|{\rm{\Psi }}(G(t))\rangle =-i{\hat{P}}_{{\mathscr{T}}(G(t))}\,H|{\rm{\Psi }}(G(t))\rangle ,$$

and provide an approximate solution of the original equation on the manifold of TT tensors of fixed rank, ${ {\mathcal M} }_{TT}$. In equation 28, ${\hat{P}}_{{\mathscr{T}}(G(t))}$ is the orthogonal projection into the tangent space of ${ {\mathcal M} }_{TT}$ at |Ψ(*G*(*t*))〉. We refer the reader to refs 59 and 66, where the explicit differential equations are derived and their approximation properties are analyzed, and to ref. 67 for a discussion of time-dependent TT/MPS in the theoretical physics literature.

Several techiques exist to compute the time evolution of TT/MPS^59, 68–70^. Here we adopt a methodology recently developed by Lubich, Oseledets and Vandereycken, which combines an explicit expression for the projector ${\hat{P}}_{{\mathscr{T}}(G(t))}$ and an extremely efficient second order split projector integrator specifically tailored to the TT format^59^. The computations presented in this paper have been performed using a code based on the software library developed by Oseledets and coworkers.

All results presented in this paper are numerically converged in the sense that the ranks of the TT cores are increased until no significant variations are observed in the solution. The dynamics at 300 K required an average value *r*_*k*_ = 40 to obtain fully converged results.
